# Susac Syndrome With Livedo Reticularis: Pathogenesis and Literature Review

**DOI:** 10.7759/cureus.27352

**Published:** 2022-07-27

**Authors:** Bahadar S Srichawla

**Affiliations:** 1 Department of Neurology, University of Massachusetts Chan Medical School, Worcester, USA

**Keywords:** susac's syndrome, branch retinal artery occlusion, senosrineural hearing loss, corpus callosum, livedo racemosa, susac syndrome, neuroimmunology, immunology, neurology, dermatology

## Abstract

Susac syndrome (SuS) is a rare autoimmune endotheliopathy that affects the retina, cochlea, and central nervous system (CNS). Even fewer cases of SuS have been reported with dermatological findings, including livedo reticularis and racemosa. The case of SuS reported here presents with encephalopathy, visual disturbances, hearing loss, and a diffuse rash on the abdomen and flank. Magnetic resonance imaging (MRI) of the brain confirmed lesions within the corpus callosum, and an audiogram revealed a unilateral biphasic sensorineural hearing loss in the right ear. A skin biopsy was completed that revealed congested dermal vessels with lymphocytic perivascular infiltrates consistent with livedo reticularis and vasculopathy. Management included intravenous methylprednisolone (IVMP) and a tapering oral dose of prednisone. The patient was also administered 1000 mg of cyclophosphamide with a two-week follow-up for a repeat infusion.

Cytotoxic T-lymphocytes (CTLs) and auto-endothelial cell antibodies (AECAs) are hypothesized to play a key role in the pathogenesis of SuS. Livedo reticularis occurs due to congestion of dermal vessels and can be both physiological and pathological in etiology. Pathological etiologies include autoimmune vasculopathies, connective tissue disorders, and drugs (catecholaminergic agents, amantadine, quinidine, etc.). A literature review of SuS cases with associated dermatologic findings is included. Five cases were identified, and neurologic manifestations, dermatologic manifestations, and interventions are described.

## Introduction

Susac syndrome (SuS) is an autoimmune condition classically defined by the clinical triad of encephalopathy, branch retinal artery occlusions (BRAOs), and sensorineural hearing loss. Susac syndrome is often referred to as an "endotheliopathy" that causes damage to the microvasculature (arterioles, capillaries, and venules). Branch retinal artery occlusions may cause vision abnormalities, and damage to the cochlear microvasculature can result in sensorineural hearing loss. Lesions in the brain also occur more frequently in the corpus callosum, causing encephalopathy [1]. Common radiographic findings include large white matter hyperintensities or "snowballs" within the corpus callosum and linear lesions called "spokes".

Susac syndrome typically afflicts young women in the range of 20-40 years of age. SuS is three times more prevalent in women than in men. Dermatologic manifestations in SuS have been reported however are exceedingly rare [2]. Here, we report a unique case of a 45-year-old man who presented with subacute onset hearing loss, vision abnormalities, acute intermittent encephalopathy, and diffuse rash (livedo reticularis) over the trunk. A review of the pathogenesis of SuS and livedo reticularis is described. An analysis of SuS cases with prominent dermatologic findings is included.

## Case presentation

**Figure 1:**
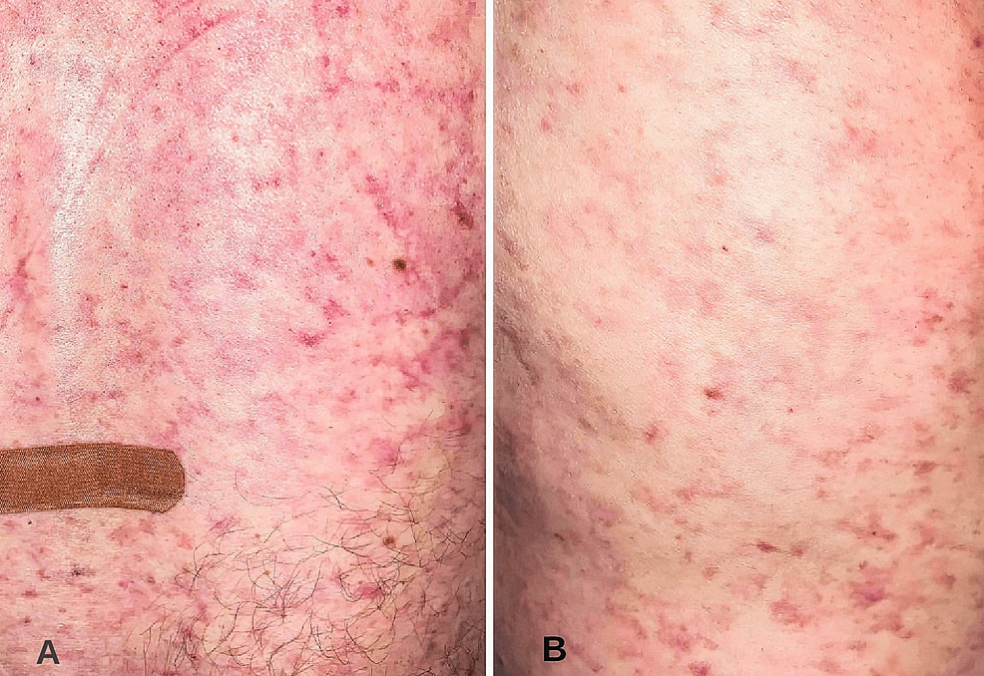
Violaceous net-like rash in an asymmetric pattern A: Rash on the lower back. B: Rash on the left flank.

A 45-year-old male with no significant past medical history presented with subacute onset hearing loss, blurry vision, and headaches. He also stated that in the past few days, he has had intermittent episodes of confusion that often require reorientation from family members and has developed a rash over his trunk. The patient does not endorse recent sick contacts, illness, recreational drug use, smoking, or alcohol abuse. The vital signs at the time of presentation were all within normal limits. A comprehensive neurologic exam was completed, and the patient was oriented to person, place, time, and situation. Further cognitive testing with word recall, serial 7s, naming, and memory were intact. An intact direct and consensual pupillary response was seen to light. However, the patient had significant deficits with peripheral vision in all four visual quadrants in both eyes. A diffuse rash was observed on the abdomen and flank of the patient (Figure 1). The rash was non-tender to the touch and blanchable.

**Figure 2:**
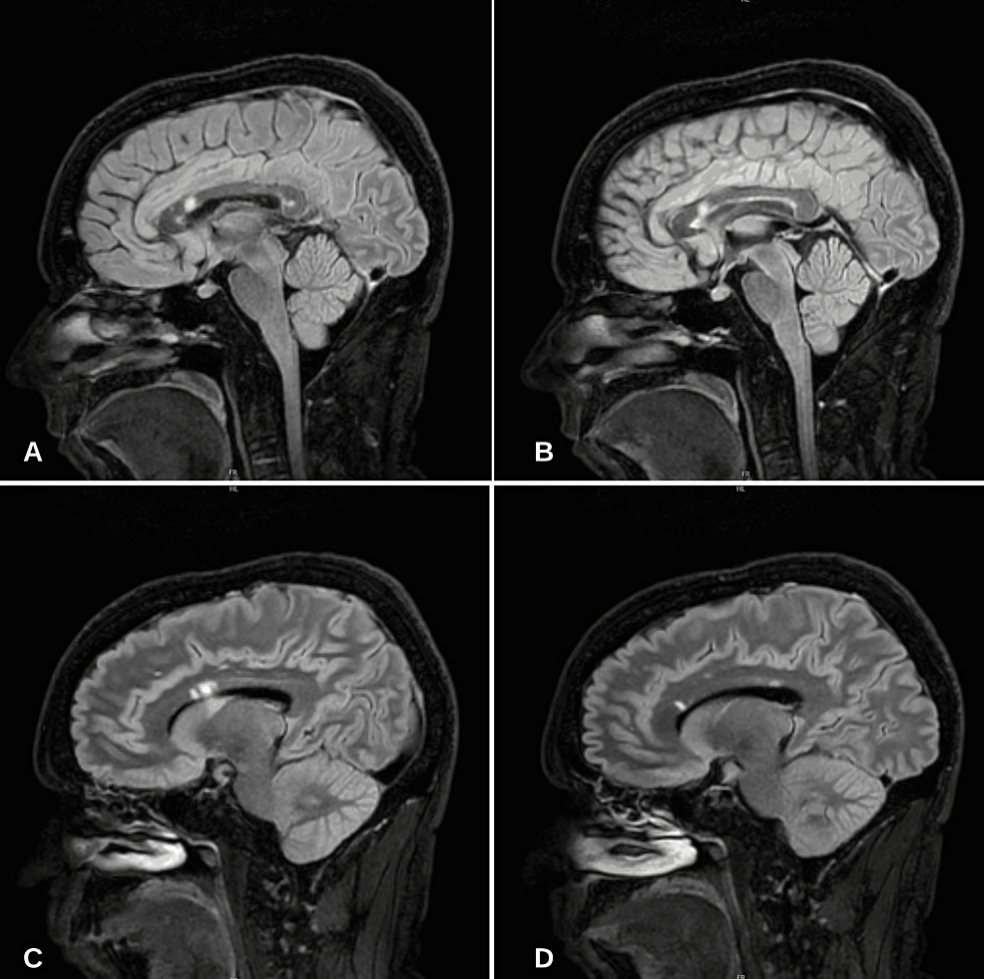
MRI T2-sagittal view revealing multiple small-to-large white matter lesions ("snowballs") and linear lesions ("spokes") A-C: Multiple large white matter lesions (snowballs) seen within the rostrum, genu, and body of the corpus callosum. D: Linear (spoke) lesion seen within the genu of the corpus callosum.

A fundoscopic exam was completed that revealed bilateral retinal flame hemorrhages and cotton wool spots. Visual acuity was 20/20 in both eyes with prescription lenses that had not changed in recent years. A comprehensive metabolic panel (CMP), complete blood count (CBC), vitamin B12, folate, thyroid-stimulating hormone (TSH), morning cortisol, copper, and iron anemia profile were within normal limits. Magnetic resonance imaging (MRI) of the brain with and without contrast was completed. Significant hyperintensities in the white matter were visualized throughout the rostrum, genu, and body of the corpus callosum (Figure 2). Cerebral angiography revealed no large vessel occlusion.

**Figure 3:**
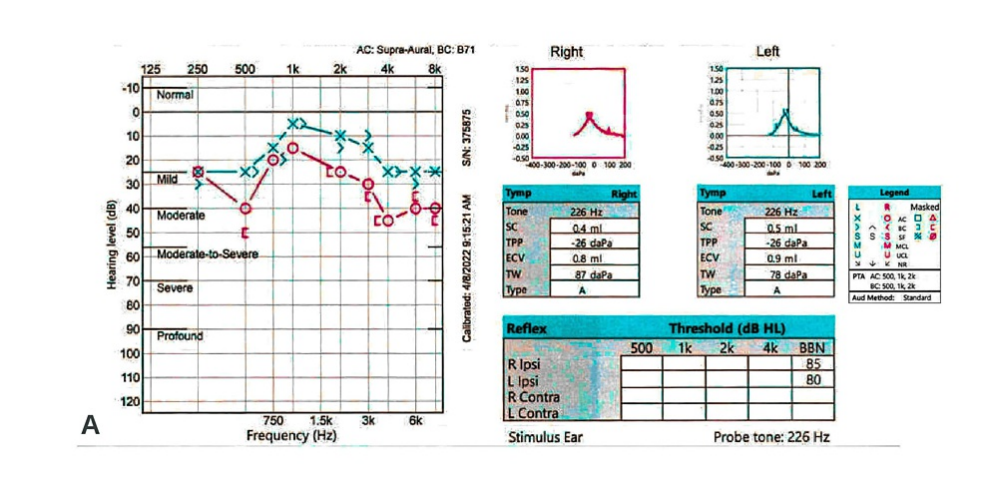
Audiogram revealing unilateral biphasic sensorineural hearing loss in the right ear SC - static compliance; TPP - tympanometric peak pressure; ECV - equivalent ear canal volume; TW - tympanometric width; AC - air conduction; BC - bone conduction; PTA - pure tone average; MCL - most comfortable loudness; UCL - uncomfortable loudness level; SF - sound field; NR - no response

The patient was negative for severe acute respiratory syndrome coronavirus 2 (SARS-CoV-2) RNA in a polymerase chain reaction (PCR) test. And the patient was vaccinated with the BNT162b2 mRNA vaccine. A complete set of blood cultures, urine cultures, and urinalysis were unremarkable. Inflammatory markers and serum autoimmune antibody panel, including C-reactive protein (CRP), erythrocyte sedimentation rate (ESR), antinuclear antibody (ANA), antineutrophil cytoplasmic antibody (ANCA), rheumatoid factor (RF), and cyclic citrullinated peptide (CCP) were negative. The patient underwent an audiogram which revealed a moderate unilateral biphasic sensorineural hearing loss in the right ear (Figure 3).

**Figure 4:**
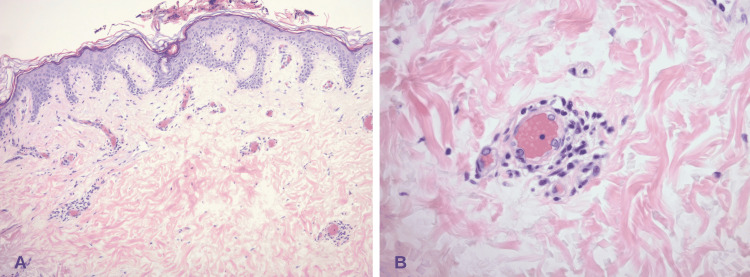
A: Ectatic and congested vessels in the superficial dermis with mild perivascular lymphocytic infiltrate; B: Congested vessel (packed with RBCs) and perivascular lymphocytic infiltrate without thrombi RBCs - red blood cells

The audiogram revealed moderate sensorineural hearing loss (SNHL) at 500 Hz, rising to within normal limits at 750-2000 Hz. Moderate SNHL is also recorded at 4000-8000 Hz in the right ear. A lumbar puncture was completed which was significant for elevated protein of 273 mg/dL (15-45), albumin of 207 mg/dL (0-35), immunoglobulin G (IgG) of 31.4 mg/dL (0-6), and IgG synthesis rate of 47.0 mg/d (<8.0) in cerebrospinal fluid (CSF). A comprehensive autoimmune and infectious panel in the CSF was unremarkable. The patient underwent a punch biopsy of the left flank that revealed mild perivascular lymphocytic infiltrate, vascular congestion, and focal vascular ectasia consistent with livedo reticularis (Figure 4).

A diagnosis of Susac syndrome was made. High-dose steroids with intravenous (IV) methylprednisolone at 1000 mg daily for five days were started. The patient was also administered a rituximab infusion; however, it was discontinued due to persistent tachycardia and the development of atrial fibrillation with a rapid ventricular response. Subsequently, 1000 mg of cyclophosphamide was administered with mesna and was well tolerated. The patient was discharged on a tapering dose of oral prednisone. At a two-week follow-up appointment, the patient was given another infusion of cyclophosphamide. He reported no further episodes of encephalopathy, resolution of the rash, improvement in hearing and peripheral vision.

## Discussion

**Figure 5:**
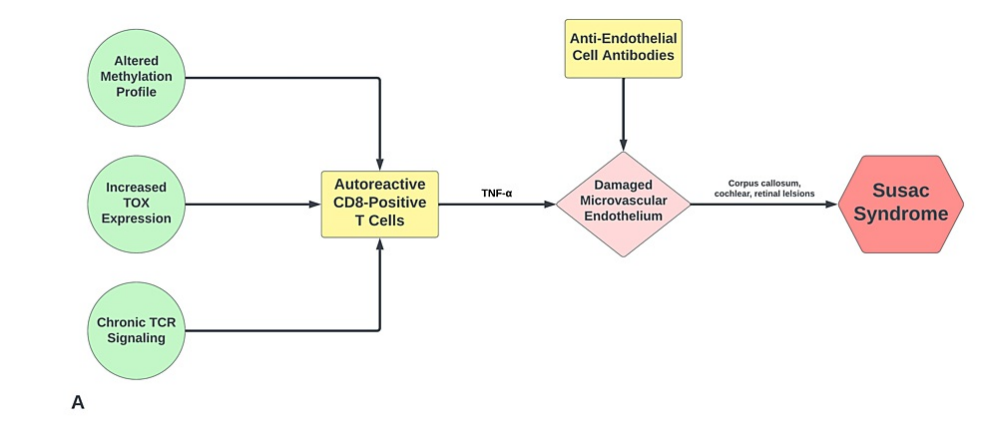
Proposed pathogenic mechanisms of Susac syndrome TOX - thymocyte selection-associated high mobility group box; TCR - T cell receptor; CD8 - cluster of differentiate 8; TNF-α - tumor necrosis factor-alpha. Image credits: Bahadar S. Srichawla

Susac syndrome (SuS) is an autoimmune endotheliopathy that disrupts the microvasculature of the brain, cochlea, and retina. The pathophysiology of SuS is believed to involve anti-endothelial cell antibodies (AECAs). A cohort study revealed that approximately 30% of SuS patients have AECAs. The presence of AECAs has a wide sensitivity to many autoimmune vasculopathies, connective tissue disorders, and inflammatory conditions and is not specific to SuS [3]. The cytotoxic cluster of differentiate 8 (CD8) positive T cells (CTLs) have also been implicated in various neuro-autoimmune conditions, including SuS. CTLs adhere to the microvasculature within the central nervous system (CNS), causing cell injury and microthrombi formation. Occlusion of these small blood vessels leads to apoptosis of neurons and various neuroglial cells, including astrocytes and oligodendrocytes. Auto-reactive CTLs are seen secondary to various mechanisms [4]. Chronic T cell receptor signaling (TCR), altered genomic methylation profile, and increased thymocyte selection-associated high mobility group box (TOX) gene expression have been implicated in the generation of autoreactive CTLs, as depicted in Figure 5 [5].

The diagnosis of SuS revolves around the identification of the clinical triad of encephalopathy, visual disturbances, and hearing loss. Some or all parts of the triad must be present with correlating objective diagnostic data to diagnose SuS. Classically, magnetic resonance (MRI) imaging of the brain reveals lesions within the corpus callosum; however, additional lesions throughout white matter are not uncommon [6]. Limbic encephalitis and flavivirus-mediated encephalitides commonly affect subcortical structures, including the basal ganglia [7-8]. Fundoscopy may reveal cotton wool spots and branch retinal artery occlusions (BRAOs). The audiogram often shows a low-frequency sensorineural hearing loss in SuS. CNS manifestations often present first, followed by visual and hearing deficits. Given the presence of sudden focal neurologic deficits and high prevalence amongst young women, SuS is often misdiagnosed as multiple sclerosis (MS). Kleffner et al. proposed diagnostic criteria for SuS stratified by definite, probable, and possible SuS based on CNS, retinal, and cochlear involvement [9]. The patient described had CNS involvement confirmed with corpus callosum lesions and sensorineural hearing loss confirmed with an audiogram. Thus, our patient has "probable SuS" based on the diagnostic criteria. The completion of fluorescein angiography (FA) revealing BRAOs and/or arteriolar wall hyperfluorescence (AWH) would complete the diagnostic triad. Approximately 13% of patients present with the complete clinical triad at presentation [10]. 

First-line treatment of SuS involves the administration of IV glucocorticoids with a transition to an oral prednisone taper. Intravenous immunoglobulins (IVIG) are also considered to be a mainstay treatment as well. Immunomodulators that are also used for treatment include mycophenolate mofetil (MM), rituximab, azathioprine, and cyclophosphamide. In a few cases, antivasospastic agents (i.e., calcium channel blockers) and antiplatelet agents have been utilized as adjunctive therapy. In this case, the patient was treated with intravenous methylprednisolone (IVMP) with an oral prednisone taper and received cyclophosphamide with good effect [11]. 

Dermatological manifestations are seen in various neurologic diseases that involve an inflammatory or autoimmune state. Pathological manifestations of livedo reticularis include antiphospholipid syndrome and amantadine use in Parkinson's disease (PD). Livedo racemosa can be seen in patients with Sneddon syndrome. Table 1 provides a summary of the clinical manifestations of various dermatologic disorders [12]. 

**Table 1:** Comparison of common dermatologic findings seen in autoimmune neurologic conditions

	Livedo racemosa	Livedo reticularis	Reticulate purpura
Pathology	Pathologic. Variable mechanism based on etiology (i.e., thromboembolic, inflammatory, etc.).	Idiopathic or physiologic.	Pathologic. Microvascular occlusions
Distribution	Asymmetrical. Non-continuous net-like pattern.	Symmetrical. Reticular pattern typically affects the limbs.	Variable symmetry. Reticular pattern.
Color	Reddish-violet	Reddish-violet	Purpuric

**Figure 6:**
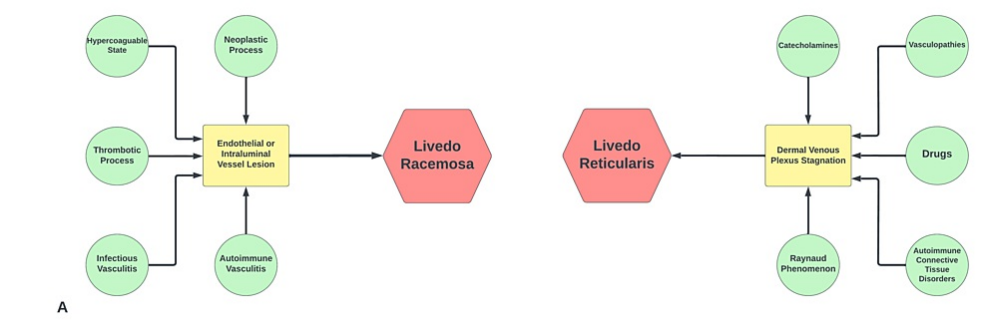
Comparison of pathophysiological mechanisms of livedo racemosa and reticularis Drugs associated with livedo reticularis include amantadine, quinidine, and catecholaminergic agents, amongst others. Image credits: Bahadar S. Srichawla

The pathophysiology of livedo reticularis involves stasis of blood within the dermal venules. This dermal stasis can occur due to vasoconstriction and is a physiological response in cold weather. It may also reflect a pathological event such as an intraluminal thrombus secondary to drugs, connective tissue disorder, or vasculopathies (Figure 6). Figure 4 shows stagnation of blood flow with congested dermal vessels filled with red blood cells (RBCs) [12]. The presence of perivascular lymphocytic infiltrates describes a livedoid vasculopathy that co-presented along with the neurologic manifestations of SuS. It can be hypothesized that immune cells affecting the microvasculature in the brain, particularly in the cochlea, retina, and CNS, are indeed the same cells mediating the cutaneous vasculopathy. However, more studies are needed to assess these claims.

Literature review

The goal of this review is to identify cases of Susac syndrome with dermatological findings to provide an overview of any associations, and interventions in the current scientific literature. A literature search was completed using PubMed/PubMed Central/Medline, Scopus, and ScienceDirect databases. The following search string was utilized ("Susac" OR "Susac's" OR "Susac syndrome" OR "Susac's syndrome") AND ("dermatology" OR "dermatological" OR "skin" OR "livedo"). Only case reports/series were included for analysis. Cases with irrelevant data, and non-diagnostic for Susac syndrome were removed. A total of five cases were identified and are shown in Table 2. 

**Table 2:** Summary table of Susac syndrome cases with dermatologic findings IV - intravenous; IVMP - intravenous methylprednisolone; IVIG - intravenous immunoglobulin; MM: - mycophenolate mofetil; MTX - methotrexate; BRAOs - branch retinal artery occlusions; ASA - aspirin

	Author	Age	Gender	Neurologic Manifestations	Dermatologic Manifestations	Interventions
1	Gertner et al. [13]	22	Male	Headache, encephalopathy, and behavioral changes	Diffuse rash of the upper extremities and chest. (Livedo reticularis)	Five days of high-dose IVMP. IVIG and rituximab
2	Engeholm et al. [14]	32	Female	Severe encephalopathy, visual abnormalities, hearing loss	A rash over trunk and extremities. (Livedo racemosa)	IVIG, MM, MTX
3	Turc et al. [15]	24	Male	Subacute encephalopathy, bilateral hearing loss, visual deficits (BRAOs)	A rash over bilateral flank and feet. (Livedo racemosa)	Three days IVMP, oral prednisolone, and ASA
4	Tashima et al. [16]	36	Male	Subacute encephalopathy, bilateral sensorineural hearing loss	Livedo reticularis	Oral prednisolone, diltiazem, and ticlopidine
5	Allmendinger et al. [17]	43	Male	Headache, encephalopathy, visual deficits	Livedo reticularis	IVIG, cyclophosphamide, glucocorticoids

Gertner et al. (2016) reported a case of a 22-year-old male with acute onset headache, altered mental status, and behavioral changes. He also demonstrated a rash on the bilateral upper extremities and chest consistent with livedo reticularis. Magnetic resonance imaging (MRI) revealed a lesion in the posterior corpus callosum, and fluorescein angiography revealed 14 branch retinal artery occlusions (BRAOs). The patient was treated with 1000 mg of methylprednisolone for five days. On day one of diagnosis, the patient received 1000 mg of rituximab, and on day two received intravenous immunoglobulin (IVIG) 500 mg/kg. On day six, the patient transitioned to oral prednisone at 1 mg/kg/day. By day ten, the patient's neurologic symptoms and the rash had resolved [13].

Engeholm et al. (2013) reported a case of a 32-year-old female with altered mental status, unsteady gait, and slurred speech. CSF studies revealed lymphocytic pleocytosis. The patient was initially diagnosed with multiple sclerosis (MS) and started with 500 mg intravenous methylprednisolone (IVMP) for five days. The patient developed an exanthema on the trunk and extremities and was diagnosed as livedo racemosa. The patient was discharged and presented six weeks later with the clinical triad of encephalopathy, hearing loss, and visual abnormalities. The patient was diagnosed with Susac syndrome (SuS) and was treated with IVMP for five days, followed by oral prednisolone. IVIG was started at 2000 mg/kg for five consecutive days. Long-term therapy was started with mycophenolate mofetil (MM) at 2000 mg/day and methotrexate (MTX) at 15 mg weekly. The patient improved, and no further relapse was noted on this regimen at a one-year follow-up [14].

Turc et al. (2011) reported a case of a 24-year-old male with subacute onset encephalopathy, gait unsteadiness, and rash over the bilateral flank and feet. MRI revealed hyperintensities in the corpus callosum, periventricular white matter, and cerebral peduncles. Fundoscopic examination revealed BRAO. Audiometry revealed bilateral sensorineural hearing loss. Histology of the skin biopsy revealed eosinophilic strands of fibrin occluding dermal peri-sudoral arterioles consistent with livedo racemosa. The patient was given 1000 mg of IVMP for three days, then oral prednisolone and aspirin. By four months, the patient had recovered completely, and his rash had resolved [15].

Tashima et al. (2001) reported a case of a 36-year-old male with subacute onset encephalopathy, visual deficits, and bilateral hearing loss. CSF studies revealed an elevated protein of 288 mg/dl and an IgG level of 16.4 mg/dl. Magnetic resonance imaging was significant for white matter hyperintensities within the basal ganglia and the internal capsule. The patient was diagnosed with MS and was started on IVMP 1000 mg/day for three days, followed by an oral methylprednisolone taper. The patient had a relapse six months later with scattered livedo reticularis on his skin, encephalopathy, ataxic gait, sensorineural hearing loss, and increased tendon reflexes. Fundoscopy revealed BRAOs in the left eye, and pure-tone audiometry revealed a bilateral asymmetric sensorineural hearing loss in low to moderate frequency tones. The patient was treated with oral prednisolone 60 mg daily for two weeks and then maintenance therapy 10 mg daily. The patient was also started with ticlopidine 300 mg/day and diltiazem 200 mg/day. Two years on these agents resulted in no further relapses and near baseline functionality [16].

Allmendinger et al. (2014) reported a case of a 43-year-old male with acute onset headache and confusion. Magnetic resonance imaging of the brain revealed T2-hyperintensities within the white matter and the central corpus callosum. CSF studies revealed elevated protein and lymphocytes without oligoclonal bands. The patient was started on IVMP followed by IVIG and was discharged on an oral prednisolone taper. The patient represented one month later with multiple focal neurologic deficits and altered mental status. An audiogram revealed sensorineural hearing loss, BRAOs on fundoscopy, and a rash consistent with livedo reticularis. The patient was treated with cyclophosphamide, IVIG, and corticosteroids with moderate improvement in deficits [17].

## Conclusions

A case of Susac syndrome (SuS) that presents with encephalopathy, headache, blurred vision, and hearing loss is described. The patient also had a diffuse rash on the flank consistent with livedo reticularis. Magnetic resonance (MR) imaging of the brain revealed lesions within the corpus callosum, impaired peripheral vision on the exam, and an audiogram revealing sensorineural hearing loss. A skin biopsy revealed dermal venous congestion and perivascular inflammation consistent with livedo reticularis and vasculopathy. The pathogenesis of SuS is described as involving cytotoxic T lymphocytes (CTLs) and anti-endothelial cell antibodies. A literature review is provided describing cases of SuS with concurrent dermatologic manifestations, most commonly livedo reticularis and racemosa. This case describes an inflammatory autoimmune process in the CNS, retina, cochlea (Susac syndrome), and dermal vessels characterized as livedo reticularis/vasculopathy.
